# Designed Y^3+^ Surface Segregation Increases Stability of Nanocrystalline Zinc Aluminate

**DOI:** 10.1021/acs.jpcc.2c07353

**Published:** 2023-02-02

**Authors:** Luis E. Sotelo Martin, Nicole M. O’Shea, Jeremy K. Mason, Ricardo H. R. Castro

**Affiliations:** † Department of Materials Science & Engineering, University of California—Davis, Davis, California95616, United States; ‡ Department of Materials Science & Engineering, Lehigh University, Bethlehem, Pennsylvania18015, United States

## Abstract

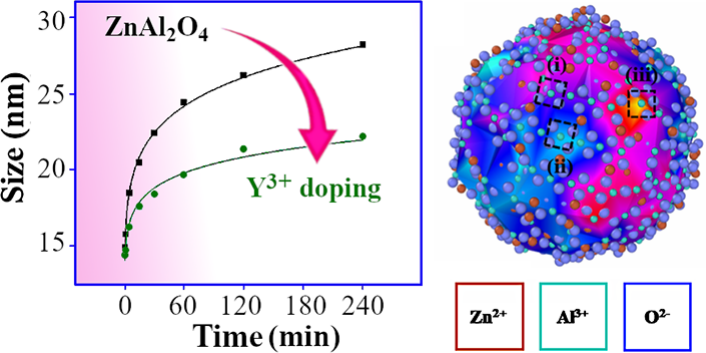

The thermal stability of zinc aluminate nanoparticles is critical for their use as catalyst supports. In this study, we experimentally show that doping with 0.5 mol % Y_2_O_3_ improves the stability of zinc aluminate nanoparticles. The dopant spontaneously segregates to the nanoparticle surfaces in a phenomenon correlated with excess energy reduction and the hindering of coarsening. Y^3+^ was selected based on atomistic simulations on a 4 nm zinc aluminate nanoparticle singularly doped with elements of different ionic radii: Sc^3+^, In^3+^, Y^3+^, and Nd^3+^. The segregation energies were generally proportional to ionic radii, with Y^3+^ showing the highest potential for surface segregation. Direct measurements of surface thermodynamics confirmed the decreasing trend in surface energy from 0.99 for undoped to 0.85 J/m^2^ for Y-doped nanoparticles. Diffusion coefficients calculated from coarsening curves for undoped and doped compositions at 850 °C were 4.8 × 10^–12^ cm^2^/s and 2.5 × 10^–12^ cm^2^/s, respectively, indicating the coarsening inhibition induced by Y^3+^ results from a combination of a reduced driving force (surface energy) and decreased atomic mobility.

## 1 Introduction

Zinc aluminate (ZnAl_2_O_4_) spinel is an excellent material to facilitate the catalysis of toluene degradation,^1,2^ hydroformylation,^3,4^ and hydrogenation^3,5,6^ because of its characteristically wide band gap and relatively high chemical and thermal stabilities.^3,7,8^ In the form of nanocrystals, the associated high specific surface areas increase the catalytic activity since the number of active sites directly scales with the available surface area if the support itself is the catalyst. Nanocrystals are equally attractive acting as a support alone since the available surfaces assist in uniformly dispersing active metals. However, the excess energies associated with nanocrystals give rise to processing challenges and limitations regarding viable operating temperatures. High surface energies lead to reduced activation energies for coarsening at the nanoscale,^9^ enabling grain growth at lower temperatures. Kinetic approaches have often been used to limit coarsening in the nanocrystalline regime,^10^ but surface thermodynamics also play a significant role in dictating the coarsening process.^11,12^ Similar to other metal oxides, zinc aluminate nanoparticles grow via Ostwald ripening,^13^ where small particles evaporate and precipitate or diffuse onto larger ones to reduce the surface energy of the system by mean grain enlargement.^14−16^ This growth mechanism is identified by its cube-root dependence on time, as shown in eq 1:
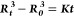
1where *R*_*t*_ is the mean particle radius at time *t*, *R*_0_ is the mean radius at the onset of growth, and *K* is a rate constant directly proportional to both the average particle surface energy and governing diffusion coefficient.^17^ For systems that undergo Ostwald ripening, this relationship highlights the significance of surface energies in hindering coarsening, a dependence that is increasingly relevant at the nanoscale due to the substantial rise in surface area.^15^

Krill et al. derived a model describing the systematic reduction of interfacial energies from dopant segregation based on the Gibbs adsorption isotherm, and this model can potentially be applied to improve thermodynamic stability at the nanoscale.^18,19^ The model describes the relationship between interfacial energy and the concentration of a segregated species, *B*, as shown in eq 2:
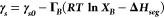
2where *γ*_*s*_ is the surface energy of the doped material, *γ*_*s*__0_ represents the surface energy of the undoped material, *Γ*_*B*_ is the solute excess at the interface, *X*_*B*_ is the concentration of the dopant, and Δ*H*_*seg*_ is the enthalpy of segregation of the dopant to the surface.^20^ The theory reflects the idea that dopants with spontaneous segregation enthalpies reduce the surface energy, thereby decreasing the driving force for coarsening.

Exploiting this concept, Hasan et al. observed limited coarsening in rare-earth doped magnesium aluminate, isostructural to zinc aluminate. After calcination at 1000 °C, the specific surface area for La-doped magnesium aluminate remained ∼50% larger than that of the undoped specimen.^21^ Surface energy measurements revealed a reduction of ∼0.3 J/m^2^ in the doped samples that was attributed to the ion surface segregation. Similar studies do not exist in zinc aluminate, but Yang et al. showed evidence of grain growth inhibition in Al-rich zinc aluminate nanoparticles compared to the stoichiometric system.^22^ Since excess Al commonly accumulates at spinel interfaces,^23−25^ the shift in zinc aluminate growth kinetics suggests that dopant segregation may induce a similar effect.

In this work, we study the effect of four different dopants [Sc^3+^ (74.5 pm), In^3+^ (80.0 pm), Y^3+^ (90.0 pm), and Nd^3+^ (98.3 pm)]^26^ on the surface thermodynamics and stability of zinc aluminate nanoparticles. All dopants are isovalent with Al^3+^ but span a range of ionic radii to systematically assess the effect of the elastic strain energies (due to the size mismatch with Al^3+^) on the surface energetics.^26^ Atomistic simulations of a 4 nm nanoparticle consistently showed preferential Y^3+^ segregation to surfaces when substituting for Al^3+^ ions.

Experimental data on surface energies measured by water adsorption microcalorimetry confirmed the simulation trends, demonstrating reduced surface energies caused by Y-doping. Coarsening studies further showed the improved stability of Y-doped zinc aluminate, with the nanoparticles exhibiting reduced grain growth compared to their undoped counterparts. Fitting the data with the Ostwald ripening model led to a better understanding of the synergistic effect of thermodynamic and kinetic effects on coarsening inhibition.

## 2 Methods and Experimental Procedures

### 2.1 Atomistic Simulations on a 4 nm Nanoparticle

The relative segregation potentials of four dopants (Sc^3+^, In^3+^, Y^3+^, and Nd^3+^) to zinc aluminate surfaces were investigated using molecular dynamics simulations of a 4 nm (3427 atoms) nanoparticle. The structure was visualized in OVITO,^27^ and all calculations were performed with the LAMMPS^28^ software using long-range Coulomb interactions and short-range Buckingham pair potentials as in eq 3:^29^
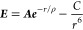
3where *E* is the potential energy of the short-range interaction between a pair of atoms; *A*, ρ, and *C* are coefficients unique to each atom pair; and *r* is the interatomic distance.^28^ Buckingham coefficients for all atomic pairs used in this study are included in Table 1; a zero for the *C* parameter indicates that the interaction is dominated by the repulsion of inner-shell electrons. The short-range interactions of all cation–cation pairs were assumed to be zero as is standard practice in the literature.^30−33^ The practical justification for this is that since the ionic radius of all of the cations is less than that of oxygen, the requirement of local charge balance means that short-range cation–cation interactions are extremely unlikely, and neglecting such interactions does not preclude acceptable fits of the physical properties of the bulk material anyway.

**Table 1:** Buckingham Pair Potentials Used for Each Cation-Oxygen Pair in the Study Derived by Grimes et al.,^31^ Busker et al.,^32^ and Migliorati et al.^34^^a^

Atom Pair	*A* (eV)	ρ (Å)	*C* (eVÅ^6^)	Reference
O^2–^–O^2–^	9548.0	0.2192	32.0	Grimes et al.^31^
O^2–^–Zn^2+^	529.7	0.3581	0	Grimes et al.^31^
O^2–^–Al^3+^	1725.2	0.2897	0	Grimes et al.^31^
O^2–^–In^3+^	1495.7	0.3327	4.3	Grimes et al.^31^
O^2–^–Y^3+^	1766.4	0.3385	19.4	Grimes et al.^31^
O^2–^–Sc^3+^	1575.9	0.3211	0	Busker et al.^32^
O^2–^–Nd^3+^	3300.1	0.2868	20.8	Migliorati et al.^34^

a All other interactions were set to zero as is standard practice in the literature.^30−33^

The particle was built by replicating a zinc aluminate unit cell and appropriately deleting atoms while maintaining a net zero charge across the particle. The resulting nanoparticle was annealed at 1000 °C for 4 ns to allow charge redistribution and surface restructuring, followed by a slow quench to absolute zero. After minimizing the quenched structure, its surface energy was calculated as the difference between the potential energy of the nanoparticle and the potential energy of a bulk structure containing the same number of stoichiometric units using eq 4:
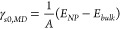
4Here *γ*_*s*__0,*MD*_ is the surface energy of the undoped 4 nm nanoparticle, *A* is the surface area of the nanoparticle, and *E*_*NP*_ and *E*_*bulk*_ are the potential energies of the nanoparticle and bulk structure with the same number of stoichiometric units.^35^

A methodology developed after Hasan et al. was used to determine the segregation potential for each dopant in the system. The method assumes that the lowest energy defect is the substitution of a trivalent dopant for an Al^3+^ atom.^35^ A series of such dopant substitutions was performed on the nanoparticles, with a single dopant atom substituted in each Al^3+^ site followed by an energy minimization.^35^ Segregation energies were estimated for each surface site in the nanoparticle by taking the potential energy difference between a substitution in the bulk and one at each surface site (defined as a site within the outer 1 Å of the nanoparticle). The average of the surface segregation energies is reported as the surface segregation energy (*E*_*seg*_) of the dopant in the zinc aluminate nanoparticle since the experimental system involves dopant concentrations beyond the dilute limit; at experimental concentrations, 10–25% of the trivalent surface sites would be occupied by Y^3+^ depending on the particle size.

Given the values of *E*_*seg*_ for each dopant, surface energies were calculated for 4 nm zinc aluminate nanoparticles doped at concentrations equivalent to those in the experimental systems using eq 5:
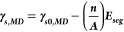
5where *γ*_*s*__,*MD*_ is the surface energy of the doped nanoparticle, *n* is the number of dopants in the system (approximately 0.5 mol % Y_2_O_3_), and *A* is the surface area of the nanoparticle. While this definition of the surface energy does not include the effect of adsorbed gas atoms that are present in experiments, the main contribution to the segregation energy is expected to be the reduction of elastic strain energy by the migration of the dopant to the free surface. Since the magnitude of this strain energy reduction should be largely independent of any adsorbed gas atoms, the simulations are expected to predict the difference *γ*_*s*__,*MD*_ - *γ*_*s*__0,*MD*_ in the surface energy between the undoped and doped samples more accurately than the absolute values of these quantities. A second reason why the surface energies calculated from simulations could deviate from the experimental data is due to complex defect chemistry not considered here (e.g., spinel site inversion, kinetically driven self-segregation within the particle originating from the space charge layer, local dipole effects of Y_2_O_3_ complexes, etc.). Nonetheless, the trends in the segregation energies should provide useful information on the relative stabilities of the four dopants in zinc aluminate.

### 2.2 Synthesis and Nanopowder Characterization

Y-doped (YZAO) and undoped (ZAO) zinc aluminate nanoparticles were synthesized via a modified coprecipitation route.^22,36−38^ Prior to synthesis, water contents were measured for each nitrate precursor to ensure stoichiometry control. Zn(NO_3_)_2_·6H_2_O and Al(NO_3_)_3_·9H_2_O (Sigma-Aldrich, >98%) were dissolved in deionized water in the metal stoichiometric molar ratio of 1:2. Hydroxides were precipitated using a 2 mol/L aqueous ammonia solution under a constant pH of 8.75 to inhibit the formation of Zn(NH_3_)_4_^2+^: precipitation of this complex limits the number of Zn^2+^ ions in the solution leading to an Al-rich environment.^22,36−38^ Precipitates were washed thrice with ethanol and dried in an oven at 80 °C for 48 h. In the case of YZAO, Y(NO_3_)_3_·6H_2_O (Sigma-Aldrich, > 98%) was also dissolved in the nitrate precursor solution to give 0.5 mol % Y_2_O_3_. Upon drying, the hydroxide precipitates were ground into fine powders. Because the coprecipitation process enables the concomitant precipitation of ions (Y, Zn, and Al) in the hydroxide form, this should lead to a homogeneous and randomized distribution of ions across the system. To test this, the dried hydroxide was analyzed in terms of phase by X-ray diffration (Figure S1) and by the chemistry distribution analyzed by energy-dispersive X-ray spectroscopy (EDX) (Scios DualBeam SEM/FIB, FEI), where the scanning electron microscope (SEM) images are obtained simultaneously (Figure S2). The results indicate even before the calcination the hydroxide already shows some crystallinity (not spinel) but no segregation of Y could be observed. The phenomenon of segregation is only expected after decomposition of the hydroxide for the crystallization of the oxide structure. The hydroxide powders were then calcined in a Thermo Scientific Lindberg/Blue M (Thermo Fisher Scientific Inc., Waltham, MA) box furnace at 550 °C for 4 h. Calcination temperatures were chosen to ensure complete crystallization while limiting grain growth.^22,36,39^

Phase analysis and crystallite size measurements were performed on both sets of powders using X-ray diffraction (XRD) on a Bruker D8 (Bruker, Billerica, MA) operated at 40 kV, 40 mA (CuKα radiation, λ=1.5406 Å). Match! software (Crystal Impact, Bonn, Germany) with reference pattern #96-900-7021 (Levy et al.^40^) was used to measure crystallite sizes for all X-ray analyses in this work. Errors associated with XRD-derived crystallite size measurements are on the order of 15–20%^41,42^ and are proportional to the true crystallite size.

As-synthesized powders were imaged using scanning transmission electron microscopy (STEM) on a JEOL-ARM300F Grand ARM (JEOL, Peabody, MA) to validate crystallite size measurements from XRD. Elemental mapping was performed on coarsened YZAO nanoparticles (900 °C, 1 h) using electron energy loss spectroscopy (EELS) to confirm Y^3+^ segregation to surfaces.

ZAO and YZAO powders were also analyzed by electron microprobe analysis (EMPA) with a Cameca SX-100 (Cameca, Gennevulliers, France) to compare Al:Zn ratios. Scans were taken at 10 different points on each sample and averaged to give Al:Zn ratios of 2.16 (±0.14) and 2.11 (±0.06) for ZAO and YZAO, respectively. These results confirmed that cationic ratios of both powders were within error of one another.

It has been well-documented that materials rich in Zn readily form carbonate species with CO_2_ and moisture in air.^43,44^ Such species could potentially impact nanoparticle coarsening behaviors (e.g., by pinning interfaces), so Fourier transform infrared spectroscopy (FTIR) was performed using a Bruker Tensor 27 (Bruker, Billerica, MA) to screen particles for Zn-rich carbonates prior to coarsening. Samples were compared to a reference pattern for zinc carbonate.

### 2.3 Coarsening Experiments

Before subjecting powders to coarsening experiments, each powder was treated in a box furnace (700 °C, 4 h, O_2_ environment) to remove residual carbonate species which could potentially affect the results. All coarsening experiments were performed within 12 h of that thermal treatment with powders being stored in a desiccator to prevent readsorption of carbonate species.

Powders were coarsened at two temperatures, 850 and 950 °C, in a Lindberg/Blue M (Thermo Fisher Scientific Inc., Waltham, MA) tube furnace for 0.5, 1, 5, 15, 30, 60, 120, and 240 min to study the effects of Y-doping on zinc aluminate’s Ostwald ripening behavior. Limited grain growth was observed due to the moderate temperatures, so all grain sizes in this study could be measured from the XRD patterns and the microstructures were confirmed with electron microscopy.

### 2.4 Surface Energy Measurements

Surface stability was evaluated for ZAO and YZAO nanoparticles (cleaned at 700 °C for 4 h under O_2_) by comparing surface energies measured by water adsorption microcalorimetry.^45−49^ This technique is comprised of a water vapor dosing system (3Flex, Micromeritics Instrument Corp., Norcross GA) attached to a differential scanning calorimeter (Sensys Evo, Setaram Inc., France). After degassing at 400 °C for 16 h and a subsequent series of three O_2_ and vacuum cycles to ensure removal of adsorbed carbonate species, samples are dosed in the 3Flex with controlled amounts of water vapor (one μmol) until surfaces are fully saturated with water. While this is happening, the calorimeter records a series of peaks associated with water adsorption occurring with each dose; when taken together with the adsorption isotherm from the 3Flex, surface energies can be calculated using thermodynamic models developed by Castro and Quach.^47^

In this study, approximately 20 mg of ZAO and YZAO powders (previously degassed at 700 °C for 4 h under an O_2_ environment in addition to the degassing described above) were analyzed by water adsorption microcalorimetry to determine surface energies. Reported surface energies represent an average across all surfaces present in the examined powder and were collected from a single experiment for each powder while errors were calculated by assuming a 0.15% uncertainty in relative pressures in addition to a 2% uncertainty in BET surface areas.^47^

## 3 Results and Discussion

### 3.1 Dopant Selection by Molecular Dynamics

**Figure 1:**
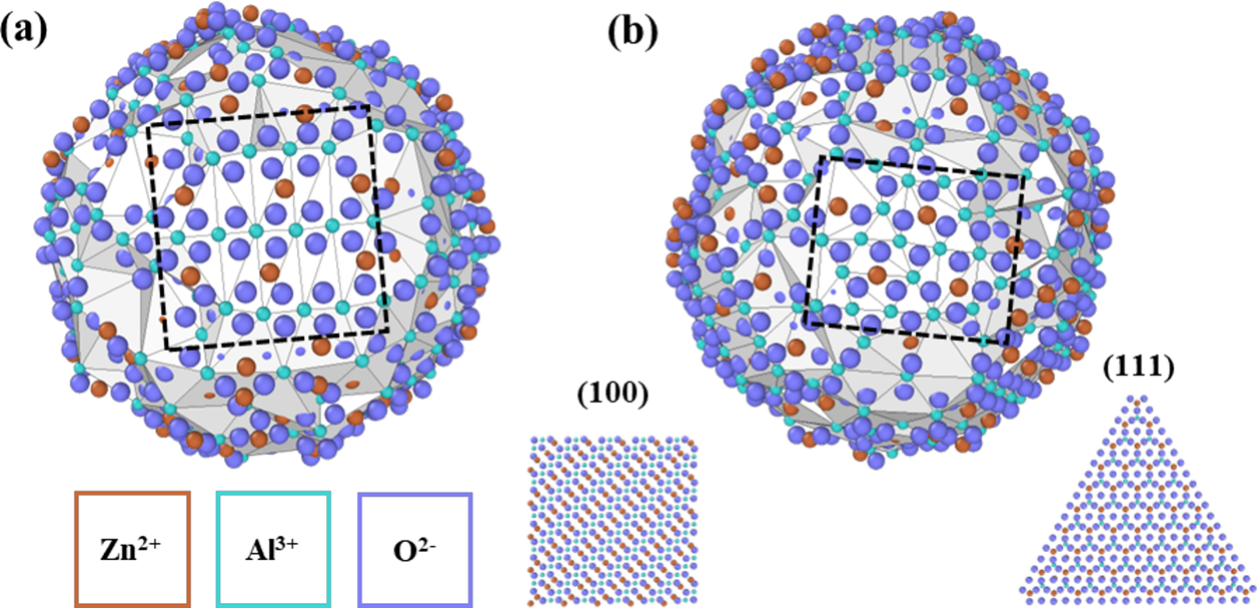
Different angles of the 4 nm zinc aluminate nanoparticle with Zn^2+^, Al^3+^, and O^2–^ represented in red (dark gray in BW), cyan (light gray in BW), and violet (mid gray in BW), respectively. Dashed lines highlight (a) (100) and (b) (111) surfaces that developed during an anneal at 1000 °C for 4 ns. Inset images show reference structures for each of these planes. A surface mesh generated in OVITO^27^ is overlaid on the nanoparticle.

Molecular dynamics (MD) simulations were used to compare the surface segregation energies of four candidate dopants (Sc^3+^, In^3+^, Y^3+^, and Nd^3+^) in zinc aluminate. A 4 nm nanoparticle of undoped zinc aluminate was built and subsequently annealed at 1000 °C for 4 ns during which it developed specific facets as indicated in Figure 1. The most prominent surface facets were on (100) and (111) planes which was expected since those have previously been identified as the lowest energy surfaces in spinel oxides.^50−52^

**Figure 2:**
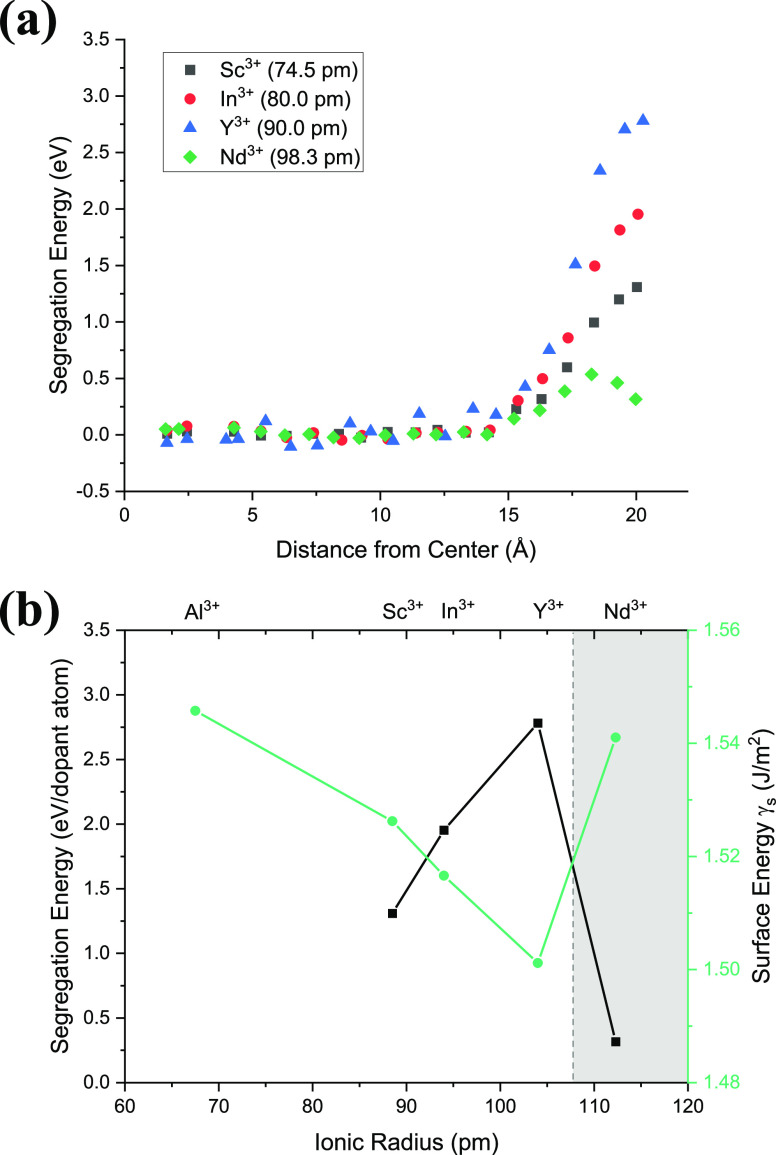
(a) Segregation energies for Sc^3+^, In^3+^, Y^3+^, and Nd^3+^ in zinc aluminate as calculated using molecular dynamics simulations of a 4 nm nanoparticle. Energies are binned by distance from the center the particle. (b) Segregation (black) and surface (green, light gray in BW) energies plotted against ionic radii for each cation type. A Y_2_O_3_ concentration of 0.5 mol % was used to estimate surface energies. Surface energies decrease as dopant ionic radius increases, though this trend breaks down for Nd^3+^.

The nanoparticle in Figure 1 was used as the starting point for simulations of dopant segregation. A single dopant atom replaced Al^3+^ ions one at a time starting from the center of the particle, with the potential energy minimized after each replacement step. Figure 2 summarizes the results, with all four dopants found to have positive segregation energies between 0.3 and 3.0 eV per dopant atom. These energies are comparable to those calculated by Hasan et al. for magnesium aluminate using two planar surfaces.^35^ The positive segregation energies indicate that zinc aluminate nanoparticles are in a lower energy state when dopants substitute at surface sites as opposed to the interior, suggesting that these dopants would all likely undergo surface segregation during synthesis. Y^3+^ had the highest segregation energy of all dopants considered at 2.78 eV with a corresponding surface energy (assuming 0.5 mol % Y_2_O_3_) of 1.50 J/m^2^, about 0.04 J/m^2^ lower than the undoped nanoparticle.

In general, the decrease in surface energies is attributed the elastic strains that arise due to ionic size mismatch in the lattice being relieved by the presence of a free surface.^35^ Unlike previous studies,^35^ the proportionality between the segregation energy and the ionic radius broke down for the largest studied ion, Nd^3+^, which showed the lowest segregation energy. No such breakdown has been reported in computational or experimental studies on interfacial segregation of dopants before this. The limited surface segregation for Nd^3+^ compared to Y^3+^ can be explained by comparing the nearest neighbors for both dopants in the bulk. Y^3+^ and Nd^3+^ have the same coordination in the bulk where both are surrounded by six O^2–^ nearest neighbors; however, the nearest neighbors are ∼0.1 Å closer to Nd^3+^ atoms than Y^3+^ atoms. This decrease in nearest neighbor distance for Nd^3+^ is associated with enhanced stability in the bulk compared to Y^3+^ which limits surface segregation. This change in nearest neighbor distance is perhaps due to the complex f orbital behavior found in Nd^3+^ which reportedly leads to a 7-fold coordination in neodymium oxide.^53^

**Figure 3:**
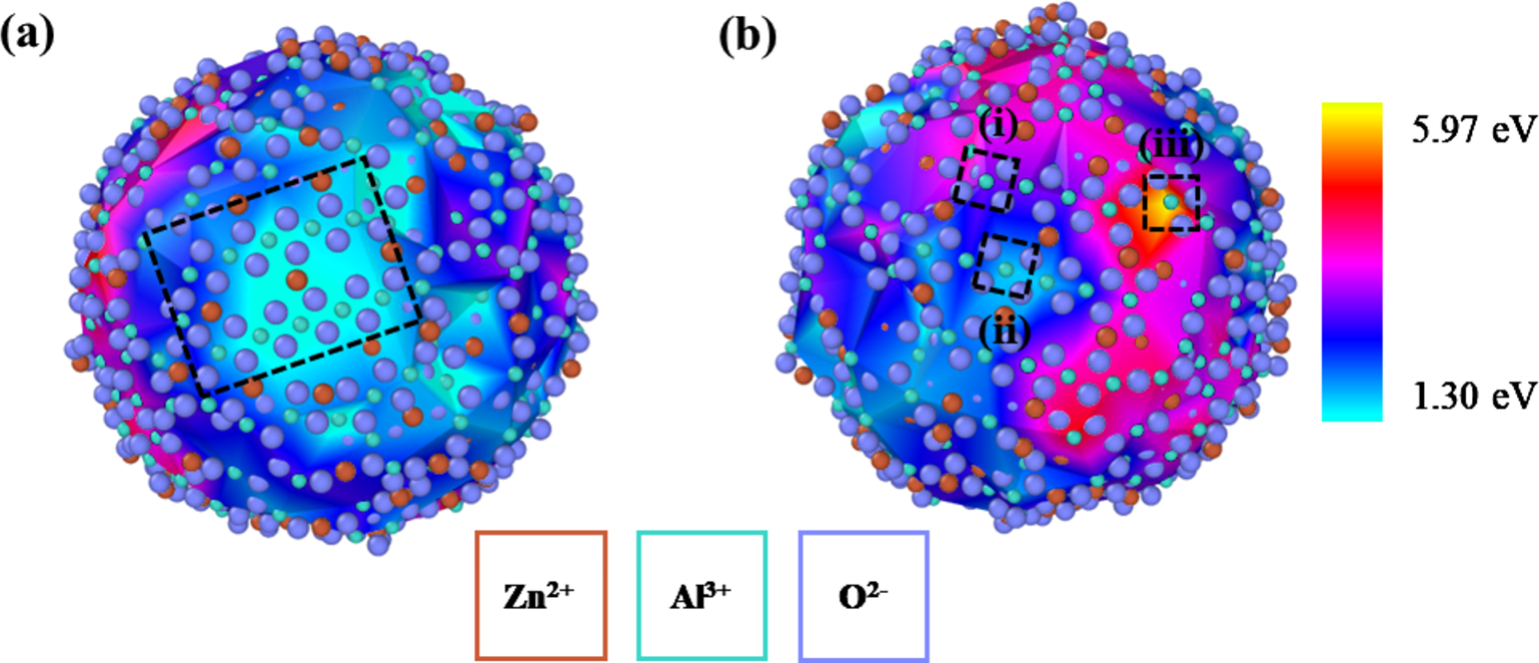
Two angles of the 4 nm zinc aluminate particle with a color gradient representing the segregation energies of Y^3+^ to trivalent surface sites. (a) A dashed (100) surface plane with relatively low segregation energies. (b) Three Al^3+^ sites with distinct energies of (i) 3.02 eV, (ii) 1.61 eV, and (iii) 5.97 eV where sites (i) and (ii) lie in a (100) facet. Color scheme is same as Figure 1.

The molecular dynamics studies also provide critical insight into the local environments that support the excess elastic strain caused by doping zinc aluminate with Y^3+^. Figure 3 shows two angles of the 4 nm zinc aluminate nanoparticle with a surface mesh color-coded to represent the segregation energy of Y^3+^ to the nearest trivalent surface sites. Figure 3a shows a (100) facet that developed during the annealing and quenching process; the red coloring that spans the entire facet indicates low segregation energies (1.3–1.5 eV) of Y^3+^. In fact, low segregation energies for highly ordered sites are found throughout the particle: Figure 3b shows another (100) facet where certain trivalent sites have low segregation energies (e.g., site (ii)).

Further examination of the facet in Figure 3b reveals a wider spread of energies than for the one in Figure 3a though. Sites (i) and (ii) are near the same facet and are surrounded by similar local environments but have segregation energies of 3.02 and 1.61 eV, respectively. This energy difference between similar sites highlights the role of nearest neighbor coordination on dopant segregation energies: the O^2–^ atoms neighboring site (ii) are near the center of the facet and highly coordinated relative to those in site (i), allowing the presence of the free surface to relieve less of the elastic strain induced by substitution of Y^3+^. This is even more apparent in site (iii), which has lower coordination than the other two sites (only three O^2–^ atoms on the surface), resulting in a segregation energy of 5.97 eV.

This relationship between segregation energies and relative positions of sites on the surface (i.e., proximity to facets and nearest neighbor coordination) predicts that Y^3+^ will preferentially segregate to sites with lower coordination. This idea has been proposed in the literature^54^ and is consistent with the fact that defects present at edges and corners directly impact nanoparticle surface energies, as demonstrated by Hummer et al. for titanium oxide.^55^ These effects tend to be less pronounced for particle sizes larger than ∼7 nm, suggesting that the segregation trends predicted here by molecular dynamics could change as zinc aluminate nanoparticles enlarge, facets develop, and the density of ledges is reduced. Explicit investigation of this size dependence is beyond the scope of the current study.

### 3.2 Synthesis and Coarsening Study

**Figure 4:**
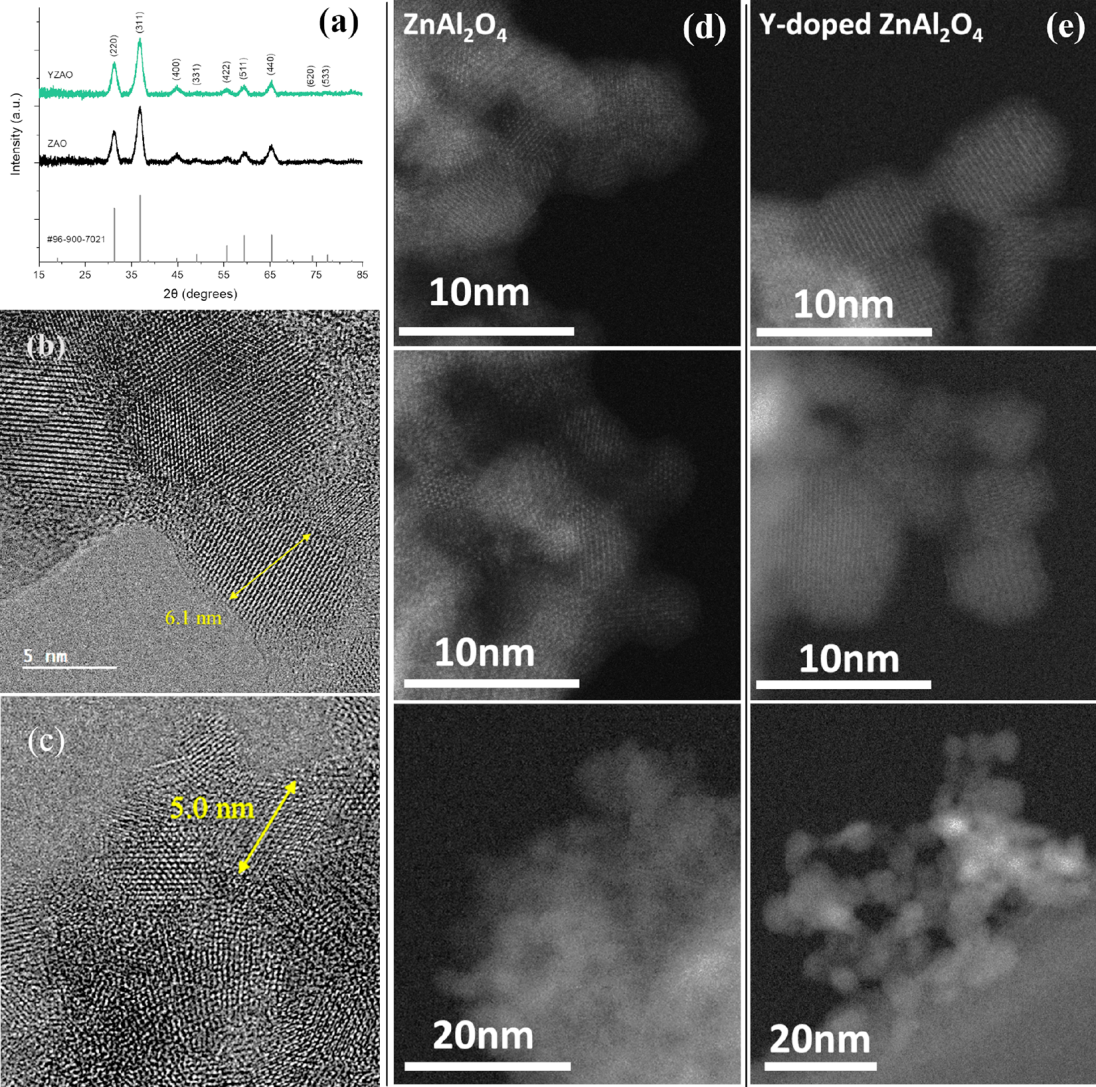
(a) X-ray diffraction patterns of ZAO (black) and YZAO (green) nanoparticles calcined at 550 °C for 4 h along with reference pattern #96-900-7021 from Levy et al.,^40^ (b) bright field transmission electron microscopy (TEM) images of ZAO nanoparticles and (c) YZAO nanoparticles. XRD peaks and TEM images both confirm particles exhibit a single spinel phase along with uniform crystallite sizes in the nanoscale. (d) STEM images of ZAO – three images vertically aligned; (e) STEM images of YZAO – three images vertically aligned.

Based on the segregation energies by atomistic simulations, Y^3+^ shows the most potential to segregate to zinc aluminate interfaces, and hence was selected for further experimental studies. X-ray diffraction patterns (Figure 4) of undoped (ZAO) and Y-doped (YZAO) zinc aluminate nanoparticles synthesized by coprecipitation revealed both sets of powders consisted of a single spinel phase. Crystallite sizes were calculated at 5.9 and 6.5 nm for doped and undoped nanoparticles, respectively. These results were consistent with exemplary TEM and STEM images of both sets of nanoparticles shown in Figure 4b–e. The images show the nanoparticles are relatively agglomerated but highly crystalline.

**Figure 5:**
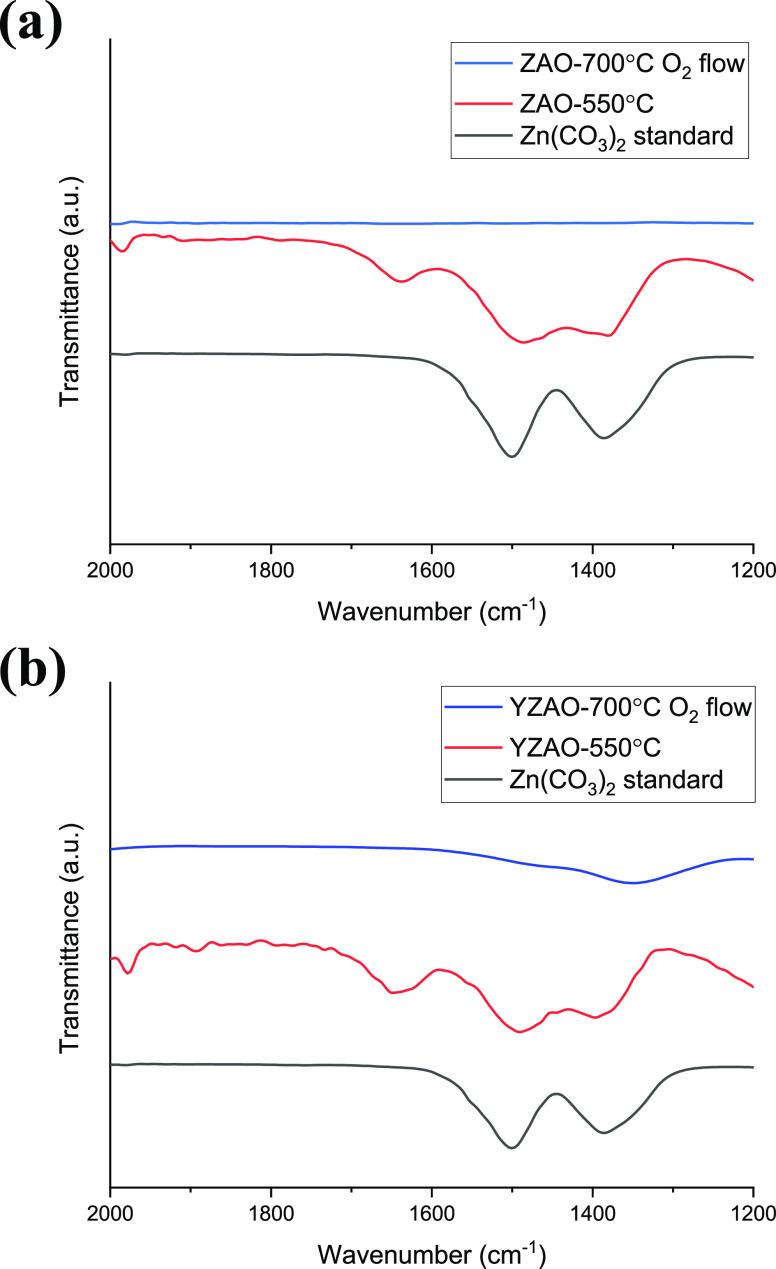
FTIR scans of (a) ZAO calcined at 550 °C for 4 h, at 700 °C under O_2_ flow for 4 h, and a zinc carbonate standard; (b) YZAO prepared under the same conditions. Zinc carbonate shows two characteristic peaks around wavenumbers 1481 and 1385 cm^–1^ which are also present in ZAO and YZAO calcined at 550 °C. A broader peak from 1630 to 1650 cm^–1^ was also found in both sets of powders at 550 °C as a result of vibrations from water adsorbed to particle surfaces.^56^

Since zinc and yttrium are likely to form stable carbonate structures, both doped and undoped nanoparticles were screened for surface carbonate species using FTIR spectroscopy prior to further studies. ZAO and YZAO prepared at 550 and 700 °C (under O_2_) for 4 h were compared to a zinc carbonate standard. Figure 5 shows that both ZAO and YZAO nanoparticles contain zinc carbonate peaks at about 1480 and 135 cm^–1^ when calcined at 550 °C. The broad peak around 1630–1650 cm^–1^ in both patterns is attributed to the vibration of adsorbed water.^51^ Each of the three peaks is absent in ZAO cleaned at 700 °C, confirming these conditions are appropriate for removing zinc carbonates in zinc aluminate. YZAO nanoparticles treated at 700 °C still showed two low-intensity broad peaks at lower wavenumbers. However, the shift in peaks’ positions indicates the carbonate groups are now only physically bonded CO_2_, absorbed during transferring of the samples from the furnace to the FTIR spectrometer.^52^ Because they are loosely bound, such carbonates should have a negligible effect on coarsening and water adsorption studies as we assume the exposure to elevated temperatures (850 and 900 °C in coarsening experiments and degassing at 400 °C for 16 h) for even short times should effectively remove these species.

**Figure 6:**
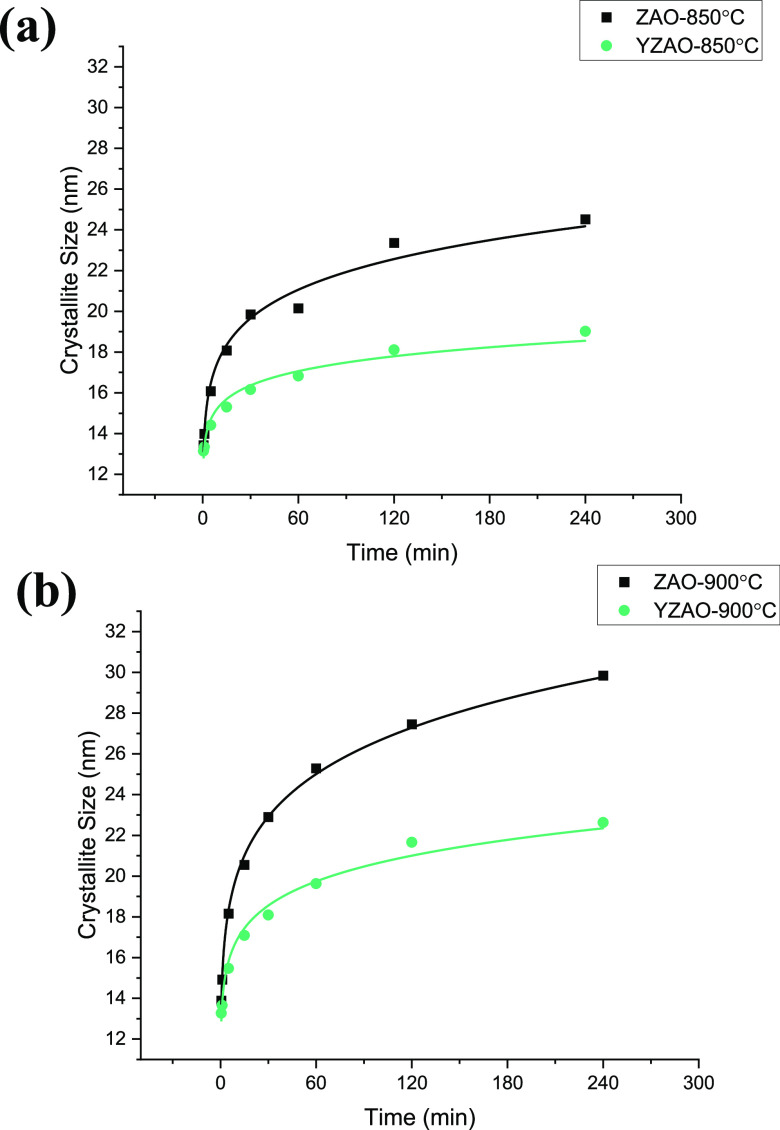
Coarsening curves at (a) 850 °C and (b) 900 °C for clean ZAO and YZAO powders with crystallite sizes estimated using XRD. YZAO particles grow less than 1 nm between 2 and 4 h at 850 °C while ZAO continues growing up until the 4-h mark, with a similar trend found in data collected at 900 °C. These results indicate doped nanoparticles undergo limited growth relative to their undoped counterparts which may be attributed to surface segregation of dopants. Estimated errors in crystallite size measurements are between 2 and 5 nm depending on coarsening time.

ZAO and YZAO nanoparticles were coarsened at 850 and 900 °C for up to 4 h to analyze the effects of doping on the coarsening behavior. ZAO and YZAO crystallite sizes were measured at 13.0 and 13.3 nm, respectively, after the annealing (cleaning) at 700 °C. Figure 6 shows typical coarsening patterns for both samples, with fast grain enlargement in the early stages of coarsening and a plateau at longer times, dependent on temperature and composition. ZAO nanoparticles coarsened to an average diameter of 24.5 nm when subjected to a temperature of 850 °C for 4 h, while YZAO only grew to 19.0 nm. Similarly, at 900 °C, ZAO underwent more growth (29.8 nm) than YZAO (22.6 nm) after 4 h. The coarsening curves at both temperatures show a difference in growth behaviors for doped and undoped nanoparticles where YZAO particles undergo limited growth, potentially due to enhanced surface stability from Y^3+^ segregation, as discussed further below.

### 3.3 Surface Stability Characterization

**Figure 7:**
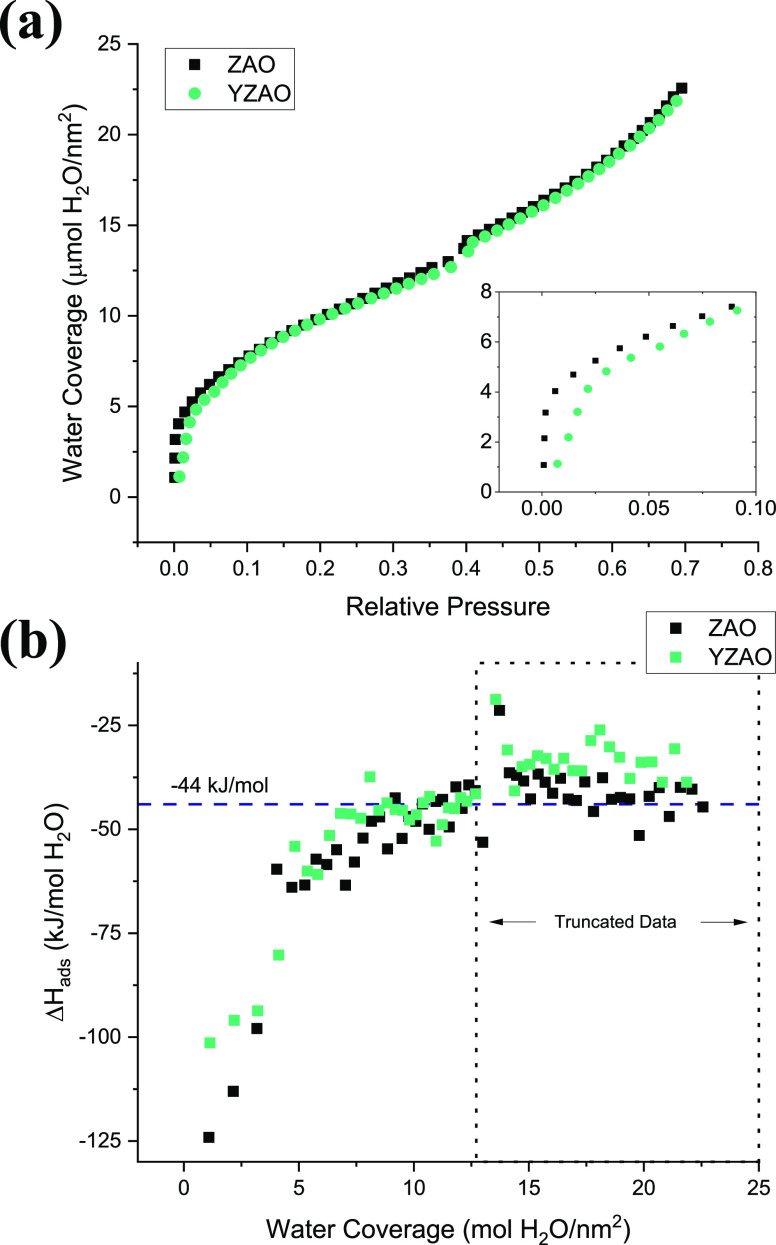
(a) Adsorption isotherm and (b) enthalpies of adsorption as a function of surface coverage for both ZAO and YZAO nanoparticles obtained via water adsorption microcalorimetry with a horizontal dashed line at the heat of liquefaction of water (−44 kJ/mol).^46^ The dotted line encloses data that were neglected for surface energy measurements due to low (in magnitude) heats of adsorption resulting from a combination of heat effects. Lower (in magnitude) enthalpies of adsorption in YZAO imply enhanced surface stability upon doping.

Anhydrous surface energies of clean ZAO and YZAO were measured using water adsorption microcalorimetry to understand the difference in coarsening behaviors and directly test the predicted reduction of surface energies. The method uses water as a probe for surface reactivity and thermodynamic models to correlate the heat of adsorption to the surface energies of the particles. Figure 7 shows the adsorption isotherm and the enthalpies of water adsorption for ZAO and YZAO. In Figure 7a, a typical type-II isotherm demonstrates water molecules adsorbed strongly to surfaces at low pressures, consistent with a chemisorption process (i.e., dissociative), with a change in slope above a relative pressure of 0.05. As shown in the inset, Y-doped samples show slightly lower slopes, consistent with lower surface reactivities. As the adsorption progresses, the curves converge as water adsorption becomes more physical (i.e., without dissociation). The small step at 0.4 is an artificial inconsistency caused by an automatic shift in the pressure gauge (equipment feature). Figure 7b shows the enthalpies of adsorption as a function of water coverage. For both samples, the water reactivity is high at low water coverages, attributed to dissociation reactions, and decreases with increasing coverage of the surface. Generally, more exothermic heats at similar coverages are observed in ZAO compared to YZAO, indicating improved surface stability (less reactivity) of Y-doped zinc aluminate nanoparticles.

After successive dosing, the enthalpies of water adsorption converged to the enthalpy of liquefaction of water which has a theoretical value of −44 kJ/mol.^46^ In Figure 7b, both ZAO and YZAO particles initially plateau at −44 kJ/mol between coverages of 10.5–12.5 mol H_2_O/nm^2^ but then continue to decrease in magnitude past this point. This decrease signals a different endothermic reaction taking place throughout the process of physical water adsorption, such as the formation of yttrium and zinc hydroxides on the surface, which is thermodynamically favorable.^58,59^ These additional processes convolute the heat effects at high coverages, making it difficult to determine the enthalpy of water adsorption. Because processes like hydroxide formation have relatively slow kinetics at room temperature and neutral pHs, we assume these reactions are negligible at low coverages and that both sets of data can be safely truncated prior to the endothermic event at 12.5 H_2_O/nm^2^ to allow for quantitative analysis of the heat of adsorption. This approach still allows for an accurate surface energy calculation, as detailed by Castro and Quach^47^ and later by Drazin and Castro.^46^ Therefore, anhydrous surface energies were calculated for both samples using the thermodynamic model for water adsorption developed by Castro and Quach.^47^ This method uses a thermodynamic description of the adsorption of water to the surfaces of particles whereby the free energy of the system is reduced as water adsorption progresses. If the bulk energy is unaffected by the adsorption process and one assumes negligible entropic and PV terms, the surface energy change can be calculated by
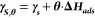
6where Δ*H*_*ads*_ is the measured heat of adsorption, *γ*_*s*_ is the anhydrous surface energy, and *γ*_*S*__,θ_ is the surface energy at a given surface coverage, θ. To minimize the contributions from the chemical potential of water, the surface energy was assumed to be equivalent to the surface energy of liquid water, 0.072 J/m^2^, at the point where the heat of adsorption converged to −44 kJ/mol. Beyond this point, adsorption peaks represent water molecules adhering to layers of water on the surface.^46^ This procedure allowed the heat of adsorption data to be used to calculate the anhydrous surface energies of both sets of nanoparticles as with other nanocrystalline oxides.^45,47,48,60^

The calculations resulted in surface energies of 0.99 (±0.02) and 0.85 (±0.02) J/m^2^ for ZAO and YZAO, respectively. The decrease in measured surface energy for YZAO relative to that of ZAO suggests that surface stability is enhanced by doping zinc aluminate with Y^3+^, further supporting the predictions from molecular dynamics simulations. The reported errors result from assuming a 0.15% uncertainty in relative pressures in addition to a 2% uncertainty in BET surface areas.^47^

**Figure 8:**
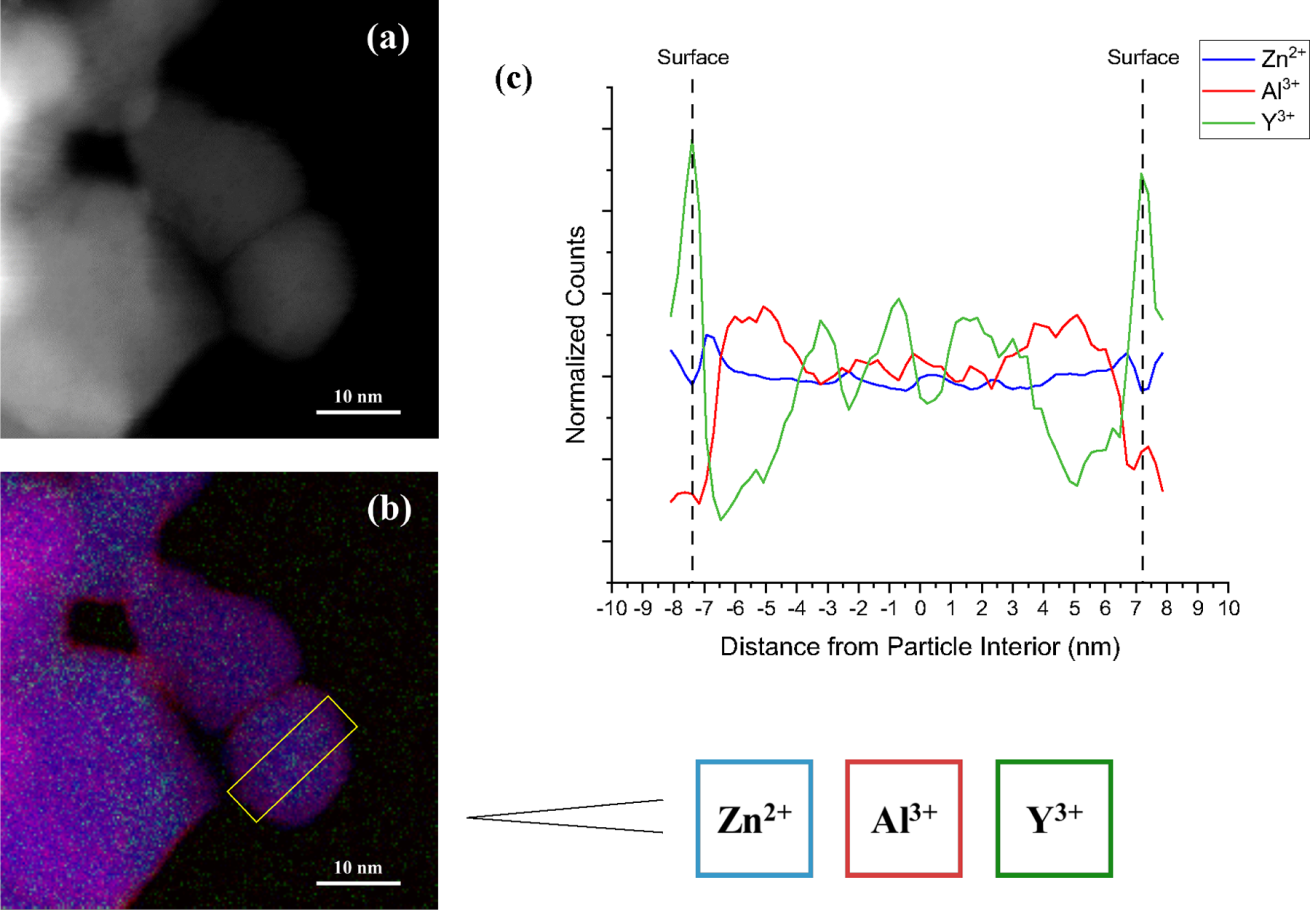
(a) Dark field TEM image of YZAO nanoparticles coarsened at 900 °C for 1 h, (b) elemental map of Zn^2+^ (blue), Al^3+^ (red), and Y^3+^ (green) taken with EELS, and (c) normalized counts of each zinc aluminate cation measured by successive box scans across the region boxed in yellow. The EELS map indicates there are two layers near the particle surfaces with different compositions than the bulk: approximately 3–6 nm away from the center of the particle, there is an increase in Al^3+^ content and a depletion of Y^3+^ followed by a spike in Y^3+^ at the surface. Al^3+^ enrichment near the particle surfaces is also visible in the colored map (b).

As simulations suggest that the cause of the reduced surface energies is dopant segregation to the surfaces, EELS mapping was performed on coarsened YZAO particles to analyze the segregation behavior of Y^3+^. Shown in Figure 8 are the EELS maps (Figure 8a) and the results from successive box scans on an individual Y-doped nanoparticle (Figure 8b). Due to its low concentration, it is difficult to visualize Y^3+^ in the EELS map, but it is apparent that Al^3+^ ions have accumulated in the vicinity of the surface as is evident from the purple shade. This is supported by the results from the box scans, which show that there is Al^3+^ enrichment (represented in red) within 3 nm of the surface edge, corroborating reports of excess Al segregating to interfaces in Al-rich spinels.^22,24,25^ The box scans further reveal that this region is depleted in Y^3+^, but as we move toward the surface edge, the spectrum detects a sharp increase in Y^3+^. This increase in Y^3+^ concentration coincides with a lowering of Zn^2+^ and Al^3+^, further validating the claims of Y^3+^ segregation to zinc aluminate surfaces. It is important to observe that the defect chemistry involved in the segregation of Y^3+^ to the surface is significantly more complex than is indicated by the molecular dynamics simulations. Namely, the presence of the surface has an effect on ion distribution in a layer roughly 3 nm thick because of the redistribution of Al^3+^ and Y^3+^. The Zn^2+^ distribution remains primarily constant, suggesting Y^3+^ and Al^3+^ share octahedral sites in the spinel structure. Presumably the large ionic radii of Y^3+^ atoms generate large lattice strains which drive them toward the surfaces and force Al^+^ to redistribute accordingly. All of that said and despite the simplified defect chemistry, molecular dynamics did effectively predict the segregation of Y^3+^.

### 3.4 Discussion

**Figure 9:**
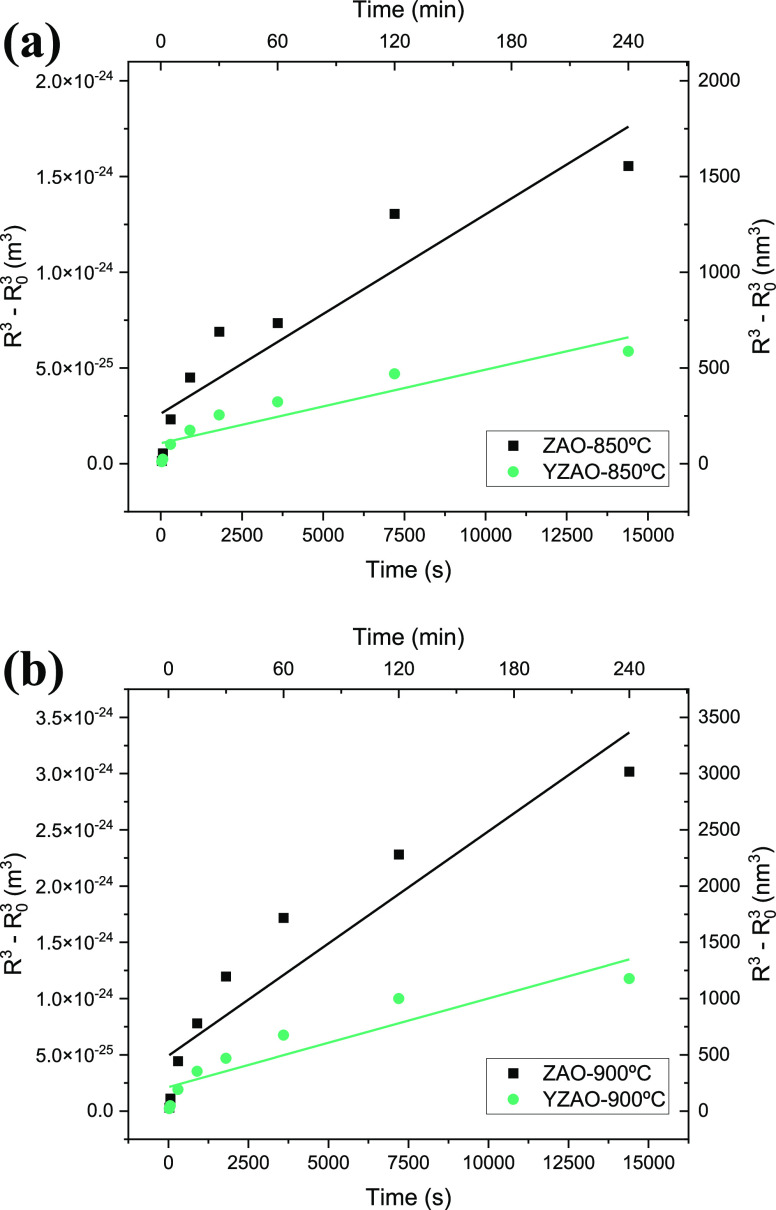
Coarsening data for both compositions at (a) 850 °C and (b) 900 °C plotted according to the Ostwald ripening equation.^17^ A linear fit is overlaid on each set of data, although crystallite sizes deviate from this fit at both short and long times.

Ostwald ripening theory states that particle growth should follow a cubic time dependence as outlined in eq 1. The equation indicates that there are two main parameters to potentially control grain enlargement: the surface energy and the diffusion coefficient. There is limited work focused on decoupling these parameters to provide a holistic understanding of coarsening control. To that end, each coarsening curve for doped and undoped zinc aluminate was plotted with a cubic dependence in Figure 9.

The linear approximation in Figure 9 overestimates crystallite sizes at short times (less than 5 min) and underestimates them at longer times. The fact that this crossover is present in all four curves suggests that either (1) the growth mechanism transitions from Ostwald ripening to a slower mechanism (e.g., migration and coalescence) as the particles enlarge or (2) the defining rate constant for Ostwald ripening changes throughout the coarsening process.

Shifts in growth mechanisms involving Ostwald ripening have been reported in the past for metal nanoparticles.^14,61^ Hansen et al. observed nickel nanoparticles in a magnesium aluminate matrix by TEM, where they noticed particle migration and coalescence even 13 s after being exposed to elevated temperatures.^14^ A transition in growth mechanisms is a consequence of a change in the relative activation energies for two mechanisms; in the case of the nickel nanoparticles, the transition from Ostwald ripening to migration and coalescence was attributed to a severe decrease in vapor pressure as the particle sizes increased.^14^ Shifts away from Ostwald ripening are more likely in metals than ceramics although since ceramic vapor pressures are much lower than in metals even at the nanoscale,^62^ meaning that ripening likely occurs via different transport mechanisms than in metals. Additionally, these types of transitions typically lead to sharp changes in the rate of crystal growth with time.^61^ The coarsening curves in Figure 6 instead show gradual changes in growth rate for both compositions at 850 and 900 °C, implying that the observed zinc aluminate growth behavior is unlikely to be due to a change in the dominant growth mechanism. Instead, the crossover in the Ostwald ripening curves is attributed to a gradual decrease in the rate constant throughout the growth process that could result from a reduced diffusion coefficient or a continuous decrease in surface energies as particles grow. Noteworthy, the images in Figure 6 indicate some agglomeration in the samples, doped or undoped. This indicates particles might be incurring in the initial stage of sintering. This process requires particle–particle contact, and while the surface energy is still the major driver, the process is significantly impacted by grain boundary energies.^11^ While this might be relevant in interpreting the morphology of the system, the initial stage of sintering does not impact particle growth significantly, and the presented discussion remains satisfactory.

For the purposes of comparing the effects of dopant segregation on the coarsening behaviors of doped and undoped zinc aluminate nanoparticles, we assume that changes in growth mechanisms and/or rate constants (i.e., surface energies) are negligible within the first 5 min of coarsening. Data before and at 5 min were used to calculate self-diffusion coefficients for both samples at 850 and 900 °C using a modified version of the Ostwald ripening rate constant developed by Lifshitz et al. which assumes atmospheric air acts as an ideal gas:
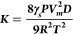
7where *γ*_*s*_ is the surface energy, *P* is the vapor pressure, *V*_*m*_ is the molar volume, *D* is the diffusion coefficient of the material, *R* is the ideal gas constant, and *T* is the temperature.^17^ Although the ideal gas assumption is questionable at room temperature, these calculations still provide a basis for comparing the kinetics of Ostwald ripening in both zinc aluminate samples. Diffusion coefficients for ZAO and YZAO along with experimental and computational surface energies are summarized in Table 2. Diffusion coefficients calculated for both undoped and Y-doped zinc aluminate are on the order of 10^–12^ cm^2^/s which agrees well with diffusion coefficients calculated for other solids at homologous temperatures.^63,64^ Juxtaposing these values makes it evident that self-diffusion is faster in undoped zinc aluminate than Y-doped zinc aluminate at both temperatures.

**Table 2:** Surface Energies and Self-Diffusion Coefficients Calculated for Y-Doped and Undoped Zinc Aluminate Nanoparticles

Dopant Content	*γ*_*s*_(J/m^2^)	*D*_850 °C_(cm^2^/s)	*D*_900 °C_(cm^2^/s)
Undoped	0.99 (±0.02)	4.8 × 10^–12^	10.1 × 10^–12^
0.5 mol % Y_2_O_3_	0.85 (±0.02)	2.5 × 10^–12^	5.1 × 10^–12^

On the atomic scale, a change in diffusion coefficient for YZAO represents a change in the rate-controlling defect for zinc aluminate self-diffusion. This defect can be identified experimentally by calculating activation energies for self-diffusion from coarsening studies and comparing them with literature values for defect formation energies. However, the present coarsening studies were limited to two temperatures which would skew such calculations. Unfortunately, to the best of our knowledge, no work has been done to identify the defects that dominate diffusion in zinc aluminate. Ting et al. found diffusion in magnesium aluminate to be controlled primarily by Schottky defects, with oxygen vacancies being the slowest diffusing species.^65^ Given that magnesium aluminate is isostructural with zinc aluminate (spinel), it is reasonable to assume defect formation energies to be similar. Hence, the dominant defect reaction in zinc aluminate is assumed to be
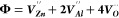
8with oxygen vacancies being the rate-controlling species. This would corroborate the trends in diffusion coefficients between doped and undoped samples: because oxygen vacancies are positively charged defects, cationic dopants in zinc aluminate (e.g., Y^3+^) would prevent the formation of oxygen vacancies and thereby limit diffusion. Furthermore, the positive charge caused by excess Y^3+^ in the surface layer would need to be accommodated by negatively charged defects.^23^ A candidate defect reaction is the delocalization of Al^3+^:
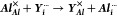
9which would likely be compensated by site inversion with the tetrahedral site, similar to magnesium aluminate.^23^ Some accumulation of Al^3+^ is already found in the elemental maps in Figure 8, supporting the idea that this series of defects could be leading to the distinct diffusive properties of ZAO and YZAO.

Although the goal of this study was to evaluate the impact of dopants on hindering coarsening in zinc aluminate, one may speculate on the effect of yttrium as a surface excess on zinc aluminate nanoparticles on their catalytic activity. It is likely the presence of Y^3+^ on the surface increases alkalinity, similarly to what is found in La-rich ZnAl_2_O_4_.^66^ Lanthanum increases the concentration of surface-isolated O^2–^ anions and OH^–^ groups increasing activity for transesterification.^66^ Although further studies are needed, the picture is consistent with the accumulation of Y^3+^ and particularly of Zn^2+^ on the subsurface, as observed in Figure 8, indicating a reduction in the local net positive charges that could be associated with the increase in the total number of basic sites.

## 4 Conclusions

This work focused on the possibility of tailoring the growth behavior of zinc aluminate nanoparticles by tuning the thermochemistry of surfaces. Molecular dynamics simulations indicated that, among several trivalent dopants, Y^3+^ was the most likely to segregate to nanoparticle surfaces. Despite the limitations of the simulations, synthesized zinc aluminate doped with 0.5 mol % Y_2_O_3_ indeed showed excess Y^3+^ located at the particle surfaces along with a more complex distribution of ions in the near-surface regions. Al^3+^ ions were depleted at the surface edge but enriched in the immediate vicinity (within 3 nm). Water adsorption microcalorimetry estimated a reduction in surface energy for doped samples consistent with the segregated Y^3+^. Coarsening studies at 850 and 900 °C demonstrated that doped (YZAO) nanoparticles exihibit more resistance to coarsening compared to undoped (ZAO) nanoparticles. This behavior results from a combination of a reduced surface energy with increasing particle size for coarsening and a decrease in the diffusion coefficient, with the latter likely stemming from the unique chemistry in the surface regions impeding the formation of the rate-limiting defect in zinc aluminate. The present work represents an important step toward controlling the coarsening behavior of nanoparticles to enable the design of stable catalytic materials.

## Supporting Information Available

Figures to support discussion. Energy-dispersive X-ray spectroscopy (EDS) elemental maps of YZAOH, the hydroxide precursor to YZAO. X-ray diffraction pattern of YZAOH along with reference pattern. (PDF)
