# Supplementary material for: Designed Y3+ Surface Segregation Increases Stability of Nanocrystalline Zinc Aluminate
Source: J Phys Chem C Nanomater Interfaces. 2023 Feb 2;127(8):4239–50. doi: 10.1021/acs.jpcc.2c07353 (PMC9986873; doi:10.1021/acs.jpcc.2c07353)
Supplement: Supplementary file 1 — jp2c07353_si_001.pdf [file jp2c07353_si_001.pdf]

Designed Y^{3+} Surface Segregation Increases Stability of Nanocrystalline Zinc Aluminate

Luis E. Sotelo Martin[1], Nicole M. O'Shea[1], Jeremy K. Mason[1], and Ricardo H. R. Castro[1,2]

[1]Department of Materials Science & Engineering, University of California, Davis, Davis, CA 95616, USA; [2]Department of Materials Science & Engineering, Lehigh University, Bethlehem, PA 18015

Supporting Information

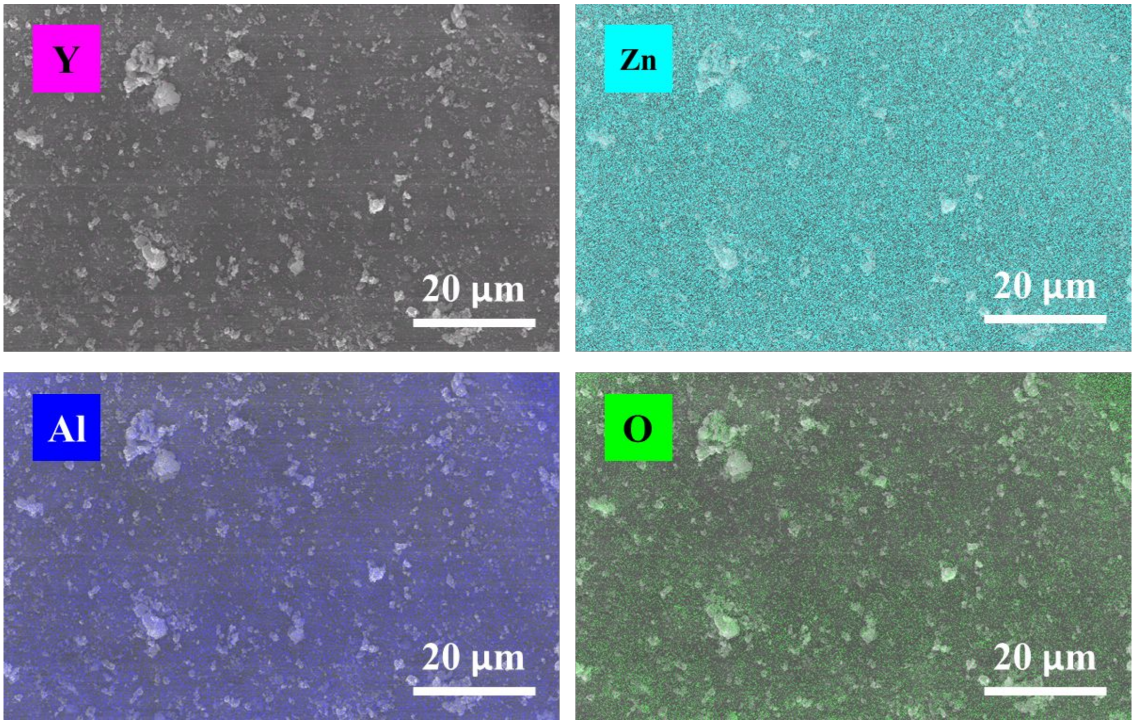

Figure S.1: Energy-dispersive X-ray spectroscopy (EDS) elemental maps of YZAOH, the hydroxide precursor to YZAO. Each element is evenly distributed throughout the image, confirming YZAOH is homogeneous.

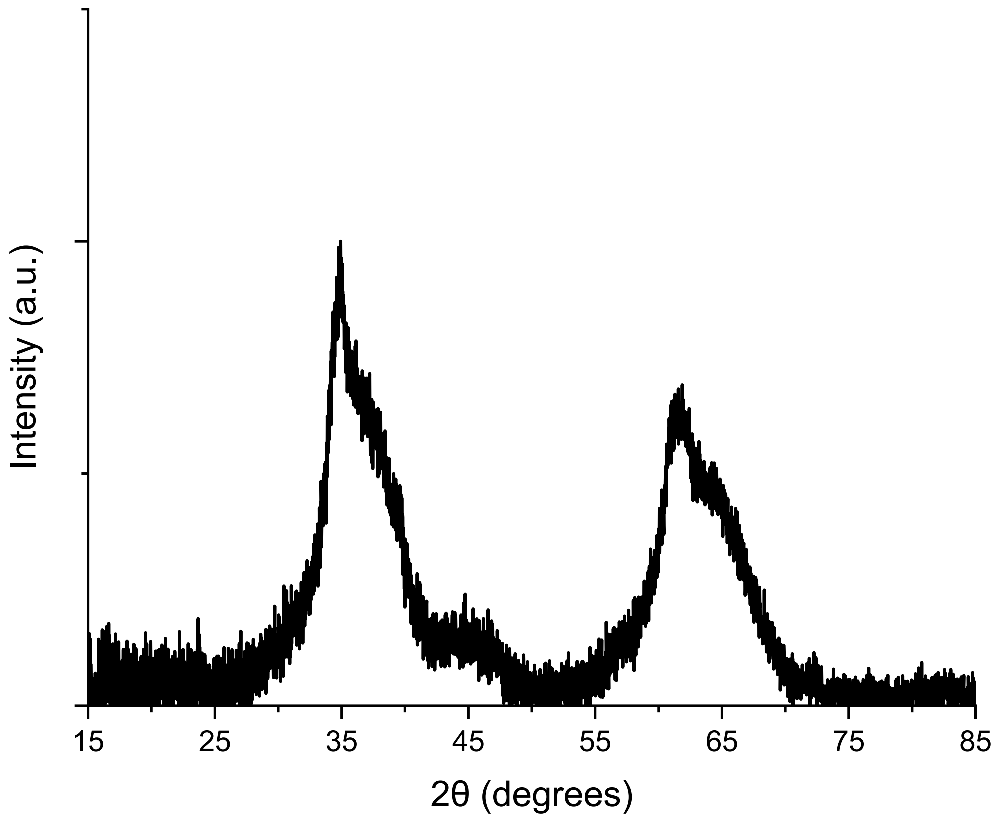

Figure S.2: X-ray diffraction pattern of YZAOH along with reference pattern #96-900-7021 from Levy et al.[1]. The diffraction pattern for YZAOH exhibits only two broad peaks around 34.9° and 61.5°, which align well with the two largest sets of peaks for zinc carbonate hydroxide as reported by Kanari et al.[2].

S.1 Supplemental References

1. Levy D, Pavese A, Sani A, Pischedda V. Structure and compressibility of synthetic ZnAl2O4 (gahnite) under high-pressure conditions, from synchrotron X-ray powder diffraction. *Phys Chem Miner*. 2001;28(9):612-618. doi:10.1007/s002690100194

2. Kanari N, Mishra D, Gaballah I, Dupré B. Thermal decomposition of zinc carbonate hydroxide. *Thermochim Acta*. 2004;410(1-2):93-100. doi:10.1016/S0040-6031(03)00396-4
